# Future optimism group based on the chronological stress view is less likely to be severe procrastinators

**DOI:** 10.1038/s41598-024-61277-y

**Published:** 2024-05-30

**Authors:** Saya Kashiwakura, Kazuo Hiraki

**Affiliations:** Graduate School of Arts and Sciences, The University of Tokyo, Meguro-Ku, Tokyo, Japan

**Keywords:** Psychology, Human behaviour

## Abstract

Previous studies have shown that procrastinators tend to disregard the future. However, the "time view" of procrastinators, including their impressions of the future, has not been sufficiently examined. Therefore, we introduced new indices, "chronological stress view" and "chronological well-being view," which treat impressions of the past, present, and future (= time view) as time-series data via stress and well-being, respectively. The results showed that the group that believed that stress did not increase as they moved into the future had a lower percentage of severe procrastinators. No relationship was found between the chronological well-being view and procrastination. This result suggests that people who are relatively optimistic about the future based on the chronological stress view are less likely to be severe procrastinators. This may suggest the importance of having a hopeful prospect in the future to avoid procrastinating on actions that should yield greater rewards in the future.

## Introduction

Procrastination is defined as "the act of putting off a task even when one knows that doing so will have maladaptive consequences"^1–3^, and has been reported to reduce people's well-being and increase their stress^4–8^. Since procrastination deeply pervades people's daily lives and affects all aspects of their lives, various factors have been examined as related to procrastination, including personality traits such as the Big Five^9–12^, cognitive and motivational factors such as self-control^13–16^, and task traits such as task aversiveness^17–19^. Among these factors, temporal characteristics have played a central role in understanding procrastination since procrastination involves putting off "present" tasks to "future" ones.

For example, in Steel & König's (2006) temporal motivation theory, procrastinators are thought to prioritize the action that provides a smaller but immediate reward over the action that should provide a larger reward in the long run because the subjective value of the reward is reduced (delay discounting) at a greater rate when the timing they get the reward is delayed^19–21^. Zhang & Feng (2020) further highlighted the impact of temporal trajectories in utility and aversiveness (e.g., the perceived task aversiveness is discounted hyperbolically when individuals postpone tasks) and constructed a temporal decision model for procrastination. Also, studies that examined the time perspective of procrastinators reported that procrastinators have weaker “future orientation” than low procrastinators and are less likely to plan and achieve future goals by actively linking the present and the future^22–24^.

It has been shown that procrastinators consistently disregard the future more than low procrastinators do and that this tendency is one of the central antecedents of procrastination. Why do procrastinators disregard the future in the first place? Although the phenomenon of future disregard has been reported in numerous studies, the underlying factors have not been clarified until now^22^.

Therefore, in this study, we examined the procrastinators' "time view" including the impression they have about the future, in order to verify the factors that form the procrastinators' future-neglectful view of time. In particular, we examined procrastinators' “time view” through stress and well-being. We focused on stress and well-being because of the abundance of previous studies examining the relationship between procrastination, and as mentioned at the beginning, it has been reported that procrastinators have higher levels of stress and lower levels of well-being^4–8^. Since stress and well-being are factors that significantly impact our lives and are generally considered negative and positive, we analyzed procrastinators' time views as comprehensively as possible with a low load for participants by selecting these two factors.

### Introducing new indicators: chronological stress view, chronological well-being view

In this study, we introduced new indices to treat procrastinators' views of time (especially their impressions of the future) as quantitatively as possible: the chronological stress view and chronological well-being view (see Fig. 5 for examples). In these indices, stress and well-being were asked at various time points in the past, present, and future (i.e., "the past 10 years," "the past 1 year," "the past month," "yesterday," "now," "tomorrow," "the next month," “the next 1 year,” and "the next 10 years.”). Then, the stress and well-being values obtained from these questions were arranged chronologically on the time axis and defined as the chronological stress and well-being views, respectively.

Most previous studies asked questions on stress and well-being by specifying a single time axis. However, in this study, we asked about stress and well-being at various times and attempted to capture procrastinators’ time views through time-series data. Our new index, the chronological stress/well-being view, is expected to capture not only the one-dimensional aspect, that is, the magnitude of stress/well-being, but also the temporal characteristics of stress/well-being (i.e., patterns of change along the time axis), and therefore expected to reflect the ideas concerning stress/well-being (e.g., stress becomes lower in the future, and well-being increases in the future).

Therefore, we decided to analyze not only the impact of absolute values of stress/well-being, but also the impact of temporal characteristics of the chronological stress/well-being view on procrastination. Specifically, chronological stress/well-being views were subjected to cluster analysis and classified into several groups based on temporal characteristics, and the proportions of severe procrastinators, middle procrastinators, and low procrastinators in each group were compared.

As mentioned earlier, studies on time perspective have shown that procrastinators tend to disregard the future. Also, mediation analyses revealed that the relationship between procrastination and future time perspective is partially explained by high stress and low positive affect^22^. Thus, one possibility is that the disregard for the future manifests itself as a belief that future conditions will worsen, such as increased stress or decreased well-being. Therefore, we hypothesized that the group that holds thoughts that lead to disregard for the future (i.e., stress increases as one moves into the future and well-being decreases as one moves into the future) would be more likely to be severe procrastinators.

The results support our hypothesis for stress and not for well-being. Specifically, we found that the proportion of severe procrastinators was lower in the group that believed that stress did not increase as one moved into the future. No relationship was found between the time series of well-being and procrastination. These results suggest that people with optimistic views of the future based on the chronological stress view may be less likely to be severe procrastinators.

## Results

### The overall mean of the chronological stress view, chronological well-being view

Since we newly introduced the chronological stress view and the chronological well-being view, we first analyzed the overall mean for each of these indices without distinguishing between severe procrastinators and low procrastinators.

**Figure 1:**
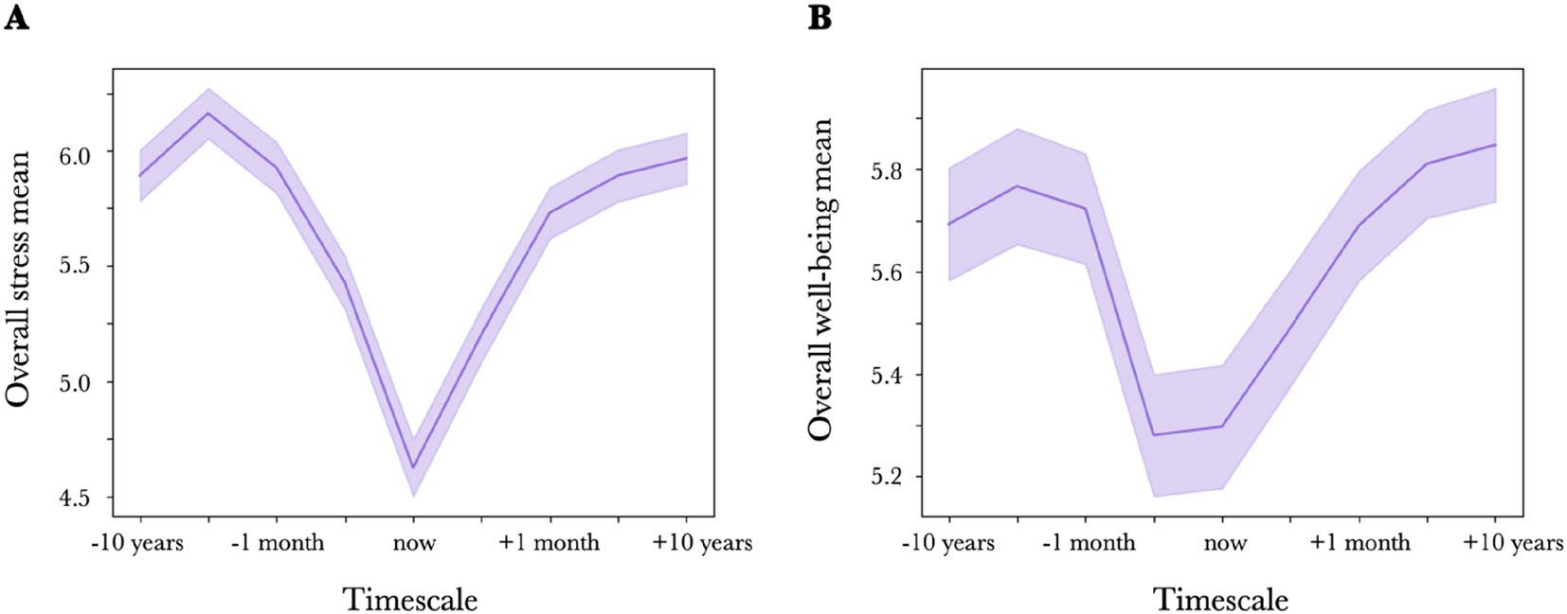
The overall mean of the chronological stress view, chronological well-being view, (**A**) Overall mean of chronological stress view; x-axis from left to right: past 10 years, past 1 year, past 1 month, yesterday, now, tomorrow, next 1 month, next 1 year, and next 10 years. The y-axis represents the stress value plotted on the x-axis time axis. (**B**) The overall mean of the chronological well-being view. The y-axis represents the level of well-being. Error bars indicate SE between participants.

As shown in Fig. 1, when stress (Fig. 1A) and well-being levels (Fig. 1B) were asked at various time points in the past, present, and future, both showed a "V-shape" with the lowest value around the "present moment" and increased as the time points moved away toward the past or future.

The means and standard deviations of stress and well-being in each temporal frame are presented in Table 1. We conducted a One-Way Repeated Measures ANOVA to examine whether there were statistically significant differences in stress/well-being levels across nine different temporal frames. For stress, the results revealed a significant effect of temporal frame on stress levels (*F*(8, 295) = 36.45, *p* < 0.001). Post-hoc comparisons using Tukey's HSD test indicated significant differences between 17/36 pairs (Table 2). For well-being, the results revealed a significant effect of temporal frame on well-being levels (*F*(8, 295) = 9.80, *p* < 0.001). Post-hoc comparisons using Tukey's HSD test indicated significant differences between 4/36 pairs (Table 2).

**Table 1:** Mean and SD for stress/well-being at each temporal frame.

	Stress		Well-being	
	Mean	SD	Mean	SD
− 10 years	5.89	0.11	5.69	0.11
− 1 year	6.16	0.11	5.77	0.11
− 1 month	5.93	0.11	5.72	0.11
Yesterday	5.43	0.12	5.28	0.12
Now	4.63	0.12	5.30	0.12
Tommorrow	5.20	0.12	5.49	0.11
+1 month	5.73	0.11	5.69	0.11
+1 year	5.89	0.11	5.81	0.11
+10 years	5.97	0.11	5.85	0.11

**Table 2:** Difference of means and adjusted p-value of post-hoc comparisons using Tukey's HSD test.

Group 1	Group 2	stress		well-being	
		Difference of Means	Adjusted P-Value	Difference of Means	Adjusted P-Value
− 10 years	− 1 year	0.27	0.757	0.07	1.000
	− 1 month	0.03	1.000	0.03	1.000
	Yesterday	− 0.47	0.088	− 0.41	0.188
	Now	− 1.27	<.001***	− 0.40	0.237
	Tomorrow	− 0.69	<.001***	− 0.20	0.938
	+1 month	− 0.16	0.985	0.00	1.000
	+1 year	0.00	1.000	0.12	0.998
	+10 years	0.07	1.000	0.16	0.988
− 1 year	− 1 month	− 0.24	0.868	− 0.04	1.000
	Yesterday	− 0.74	<.001***	− 0.49	0.056
	Now	− 1.54	<.001***	− 0.47	0.076
	Tomorrow	− 0.96	<.001***	− 0.28	0.718
	+1 month	− 0.43	0.151	− 0.08	1.000
	+1 year	− 0.27	0.757	0.04	1.000
	+10 years	− 0.20	0.952	0.08	1.000
− 1 month	yesterday	− 0.50	0.049*	− 0.44	0.119
	now	− 1.30	<.001***	− 0.43	0.155
	tomorrow	− 0.72	<.001***	− 0.23	0.870
	+1 month	− 0.20	0.952	− 0.03	1.000
	+1 year	− 0.03	1.000	0.09	1.000
	+10 years	0.04	1.000	0.13	0.997
Yesterday	Now	− 0.80	<.001***	0.02	1.000
	Tomorrow	− 0.22	0.903	0.21	0.926
	+1 month	0.30	0.619	0.41	0.198
	+1 year	0.47	0.088	0.53	0.024*
	+10 years	0.54	0.022*	0.57	0.011*
Now	Tomorrow	0.58	0.010**	0.19	0.954
	+1 month	1.10	<.001***	0.39	0.248
	+1 year	1.27	<.001***	0.51	0.034*
	+10 years	1.34	<.001***	0.55	0.016*
Tomorrow	+1 month	0.53	0.029*	0.20	0.944
	+1 year	0.69	<.001***	0.32	0.528
	+10 years	0.76	<.001***	0.36	0.370
+1 month	+1 year	0.16	0.985	0.12	0.998
	+10 years	0.24	0.868	0.16	0.986
+1 year	+10 years	0.07	1.000	0.04	1.000

*p < 0.05, **p < 0.01, ***p < 0.001.

Since no studies have asked about stress and well-being levels at various time points in this way, the study’s first finding was that the overall mean of the chronological stress view and chronological well-being view shows a pattern that could be subjectively labeled as V-shape.

### Classification of the chronological stress view and chronological well-being view by procrastination score

Before moving on to the analysis of temporal characteristics of the chronological stress/well-being views, we first conducted a basic analysis that would strongly capture the impact of the magnitude of stress/well-being in the chronological stress/well-being views on procrastination.

Based on the procrastination scores of the Pure Procrastination Scale, we defined the top 25% as severe procrastinators, the bottom 25% as low procrastinators, and the middle 50% as middle procrastinators. We then plotted the chronological stress and well-being views separately for severe and low procrastinators.

**Figure 2:**
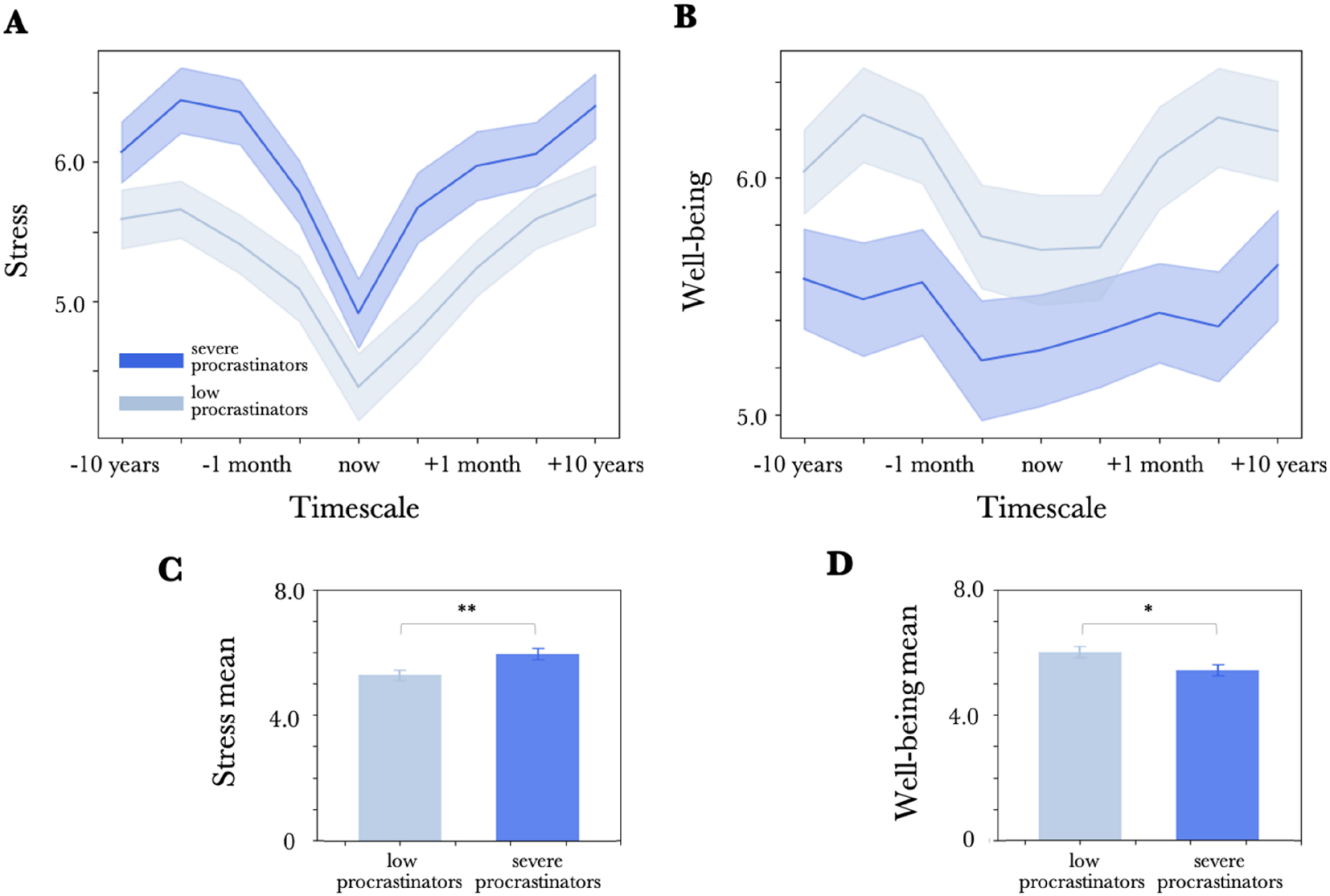
Classification of the chronological stress view and chronological well-being view by procrastination score, (**A**) Comparison of the chronological stress view between severe procrastinators (top 25% procrastination score, dark blue) and low procrastinators (bottom 25% procrastination score, light blue). The x-axis shows, from left to right, the past 10 years, past 1 year, past 1 month, yesterday, now, tomorrow, next 1 month, next 1 year, and next 10 years. The y-axis is the stress score. (**B**) Comparison of chronological well-being view between severe procrastinators (top 25% of procrastination score, dark blue) and low procrastinators (bottom 25% of procrastination score, light blue). The y-axis is the well-being score. (**C**) Comparison of overall mean stress scores between procrastinators (top 25% procrastination score, dark blue) and low procrastinators (bottom 25% procrastination score, light blue). The overall mean of the stress values was calculated within each subject by ignoring the differences in the time axis, and then the within-group mean was calculated. (D) Comparison of overall mean well-being between procrastinators (top 25% of procrastination scores, dark blue) and low procrastinators (bottom 25% of procrastination scores, light blue). Error bars indicate SE between participants for each group. (*p < 0.05, **p < 0.01).

Although two-tailed t-tests with multiple comparison correction (Bonferroni) at each time point showed no significant differences except for stress at “the past month” and well-being at “the next 1 year,” we found an overall tendency of stress being higher in the severe procrastinator group (Fig. 2A) and well-being being lower in the severe procrastinator group (Fig. 2B) across all time points (“stress” the past 10 years: *t*(69) = 1.57, *p* = 0.12, the past year: *t*(69) = 2.53, *p* = 0.01, the past month: *t*(69) = 3.02, *p* = 0.003, yesterday: *t*(69) = 2.10, *p* = 0.04, now: *t*(69) = 1.52, *p* = 13, tomorrow: t(69) = 2.64, *p* = 0.009, the next month: *t*(69) = 2.32, *p* = 0.02, the next 1 year: *t*(69) = 1.49, *p* = 0.14, the next 10 years: *t*(69) = 2.03, *p* = 0.05, “well-being” the past 10 years: *t*(69) = -1.65, *p* = 0.10, the past year: *t*(69) = -2.52, *p* = 0.01, the past month: *t*(69) = -2.09, *p* = 0.04, yesterday: *t*(69) = -1.57, *p* = 0.12, now: *t*(69) = -1.27, *p* = 0.21, tomorrow: *t*(69) = -1.14, *p* = 0.26, the next month: *t*(69) = -2.13, *p* = 0.03, the next 1 year: *t*(69) = -2.82, *p* = 0.005, the next 10 years: *t*(69) = -1.80, *p* = 0.07. , corrected alpha = 0.00556). Figure 2 C-D shows the results of calculating the within-group means for the severe procrastinators and the low procrastinators, after each subject’ overall means for stress and well-being were calculated by ignoring the differences along the time axis (x-axis). We conducted two-tailed two-sample t-tests to compare the within-group means, and the results showed that the severe procrastinators had significantly higher stress (*t*(70) = 2.78, *p* = 0.006, Fig. 2C) but lower well-being (*t*(70) = -2.30, *p* = 0.02, Fig. 2D) compared to low procrastinators.

These results replicate previous research, as previous studies have shown that procrastinators have higher stress and lower well-being. At the same time, it was newly found that the same tendency is reproduced no matter what timeframe is used, even if the question is a prediction for the future.

### Clustering analysis of the chronological stress and chronological well-being views

In the above analysis, we have confirmed that our study reproduces the tendency reported in previous studies that the magnitude of stress and well-being matters in procrastination. However, the above analysis did not reveal any differences between procrastinators and low procrastinators regarding factors encompassing temporal characteristics, such as patterns of change along the time axis (graph shape). Therefore, further analysis was conducted to extract the influence of individual time-series patterns, an essential aspect of the time view, by eliminating the influence of the magnitude of stress/well-being as much as possible.

In order to extract the influence of the patterns of the time-series data, we first standardized each individual's data to minimize the influence of the magnitude of stress/well-being (see Data analysis in Methods for details). Then, we categorized the chronological stress and chronological well-being views by clustering analysis using the (Dynamic Time Warping (DTW) method. The main reason for choosing cluster analysis is its ability to handle the dynamic nature of time-series data. Unlike latent profile analysis (LPA), typically used to identify latent groups within cross-sectional data based on underlying probability distribution assumptions, cluster analysis is suited to analyze patterns and trajectories in time-series data, this time stress/well-being along nine-time axes.

In conducting the clustering analysis, the number of clusters was first examined using the elbow method, and the number of clusters was determined to be four in this analysis (see Supplementary Information).

**Figure 3:**
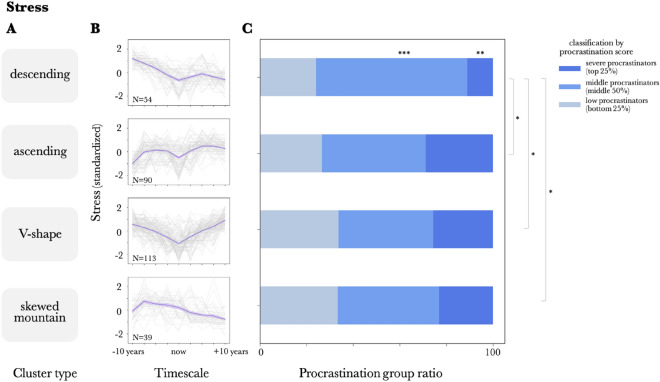
Clusters of the chronological stress view and its relationship with procrastination, (**A**) Labels of clusters for the chronological stress view (standardized). (**B**) Clusters of the chronological stress view (standardized). The dark purple line represents the within-cluster mean, the error bars represent the SE between participants within a cluster, and the light gray line represents the data for individual participants. (**C**) Comparison of severe procrastinators (top 25% procrastination score, dark blue), low procrastinators (bottom 25% procrastination score, light blue), and middle procrastinators (middle 25% procrastination score, middle blue) ratios across clusters of chronological stress view. (*p < 0.05, **p < 0.01, ***p < 0.001).

**Figure 4:**
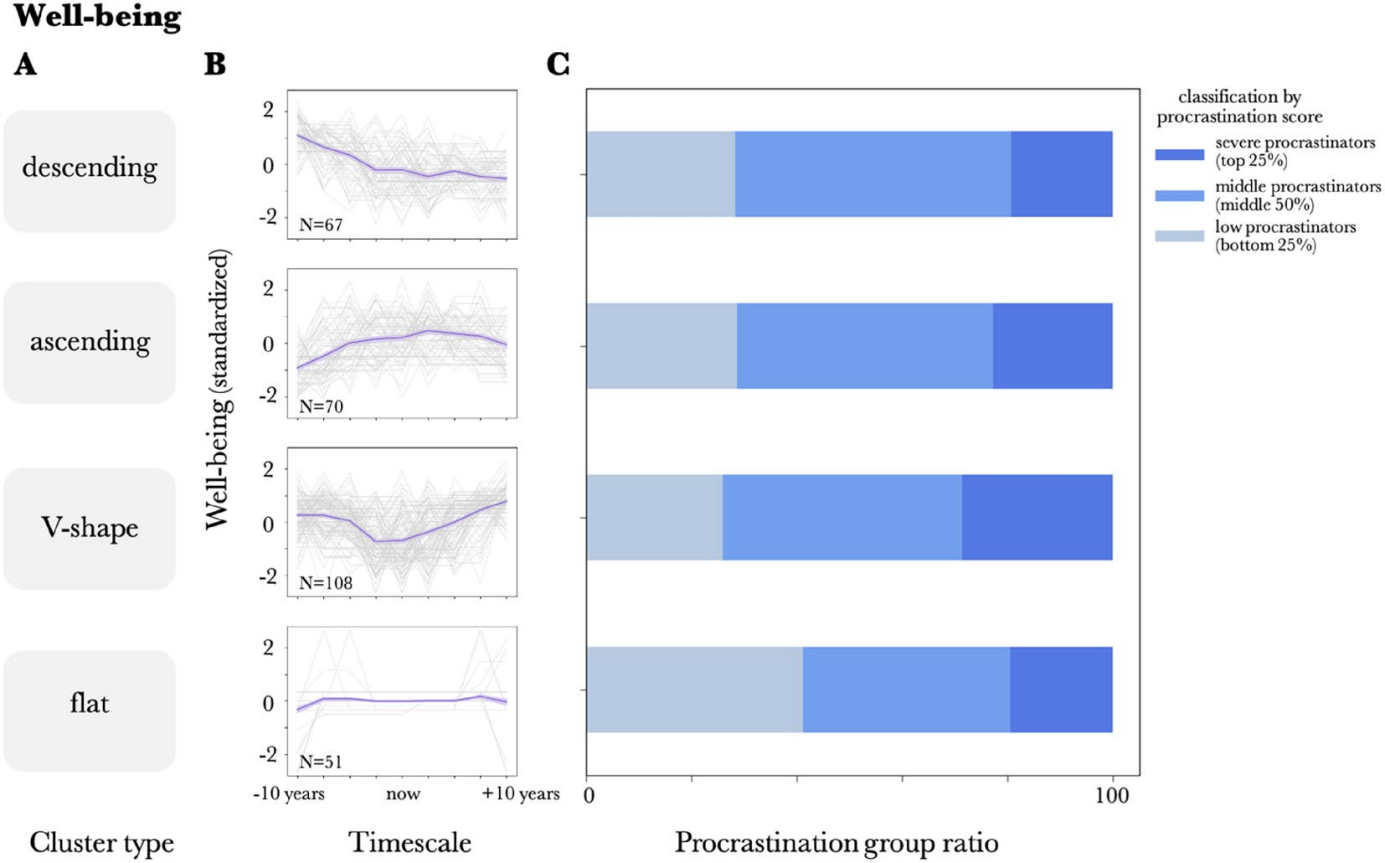
Clusters of the chronological well-being view and its relationship with procrastination, (**A**) Labels of the clusters for chronological well-being view (standardized). (**B**) Clusters of the chronological well-being view (standardized). The dark purple line represents the within-cluster mean, the error bars represent the SE between participants within a cluster, and the light gray line represents the data for individual participants. (**C**) Comparison of severe procrastinators (top 25% procrastination score, dark blue), low procrastinators (bottom 25% procrastination score, light blue), and middle procrastinators (middle 25% procrastination score, middle blue) ratios across clusters of chronological well-being view.

As shown in Fig. 3 A-B and Fig. 4 A-B, the clustering analysis revealed that both chronological stress and chronological well-being views had descending, ascending, and V-shaped types, with stress having a skewed mountain type as the fourth cluster (Fig. 3 A-B) and well-being having a flat type as the fourth cluster (Fig. 4 A,B). The results indicate that, although the chronological stress and well-being views show a V-shape when averaged over the entire sample, they can be divided into several clusters.

- Descending: Stress/well-being decreases as one moves from the past to the present and into the future.
- Ascending: Stress/well-being increases as one moves from the past to the present and into the future.
- V-shape: Stress and well-being are the lowest in the present and increase as one moves away from the present.
- Skewed Mountain: Stress/well-being reaches its highest value at a point in the past and then declines toward the future.
- Flat: Stress/Well-being is constant

### Clusters of the chronological stress and chronological well-being views and the procrastination levels

To verify the influence of the types obtained from the cluster analysis on the tendency to procrastinate, the proportions of the severe procrastinators (top 25% procrastination score), middle procrastinators (middle 50%), and low procrastinators (bottom 25%) for each type were calculated and compared using a chi-square test.

The results showed that there was a significant difference in chronological stress view (χ2(6) = 18.08, *p* = 0.006), between "descending" and "ascending" (*p* = 0.014), between "descending" and "V-shape" (p = 0.01), and between "descending" and "skewed mountain" (*p* = 0.02). A residual analysis revealed significant differences in the severe procrastinators (*p* = 0.002) and middle procrastinators group (*p* < 0.001) in the descending type (Fig. 3C). No significant relationship was found between the chronological well-being view and procrastination (χ2(6) = 9.05, *p* = 0.17; Fig. 4C).

The significantly lower percentage of severe procrastinators in the descending clusters indicated that the group who believed that stress would decrease as they moved into the future, or at least not increase more than in the past, was less likely to be severe procrastinators.

## Discussions

In this study, we introduced new indices, chronological stress and chronological well-being views, in which we asked about stress/well-being levels at various time points and attempted to examine the relationship between procrastinators' time views (impressions of the past, present, and future) and procrastination. We found that the procrastinators who believed that stress would decrease (or at least not increase more than in the past) as they moved into the future were less likely to be severe procrastinators.

Although this study revealed that those who believed that stress would decrease as they moved into the future were less likely to be severe procrastinators, the relationship between procrastination and disregard for the future could have been manifested in other patterns as well (i.e., The groups who believe that stress will decrease in the future or well-being will increase in the future, i.e., those who have an optimistic view of the future, have a lower percentage of severe procrastinators or a higher percentage of low procrastinators. The group that believes that stress will increase in the future or the group that believes that well-being will decrease in the future, i.e., the group that has a pessimistic view of the future, have a higher percentage of severe procrastinators or a lower percentage of low procrastinators.). However, only one of the eight possible patterns emerged in this study. These findings suggest that stress-based optimistic view of the future plays a significant role in procrastination, especially in determining whether or not a person becomes a severe procrastinator. Further research is needed to investigate the causes of these asymmetric results.

Previous studies have shown that procrastinators tend to disregard the future^20,22^. Although direct verification is required to confirm this finding, it is possible that procrastinators have a pessimistic view of the future and, therefore, disregard it. This may suggest the importance of having a hopeful prospect in the future to avoid postponing actions that should yield greater rewards in the long run. Direct approaches and intervention studies are needed to test these possibilities, such as examining the relationship between future orientation^25^, chronological stress view, and procrastination.

In this study, we acquired a new finding that the overall mean of the chronological stress view is "V-shaped." One of the reasons for this result may be that the present stress level was asked first in our experiment and thus became the reference point for the following questions with different time axes. However, even if the value of the present stress level becomes the reference point, it cannot be the reason for the lowest score. Therefore, the appearance of the V-shaped data requires further examination. It is also necessary to verify whether the same tendency would be obtained if other methods measured the chronological stress view.

In our study, we have focused on the “future” in particular. However, it should be noted that we measured stress/well-being on multiple temporal frames spanning the past, present, and future, and categorized chronological stress/well-being into several groups based on this continuous time-series data. In other words, "future orientation" in this study is not defined in terms of the future alone but based on the relationship between the past and the present. Therefore, future research should focus not only on the future but also on the past and present. Past studies using the time perspective scale have shown that procrastination has a small but significant positive association with the present time perspective^22^ and that procrastinators have a higher past positive past positive orientation and lower past negative orientation^23^. Based on these findings, we should comprehensively examine the relationship between procrastination and the past, present, and future.

Conventional studies on procrastination have argued simply that procrastinators are more stressed and less happy. However, this study found that not only the magnitude of stress but also the time-series stress patterns affect procrastination. Therefore, it can be said that this study shows for the first time the multilayered effect of stress on procrastination. It should be noted that the magnitude of stress and well-being values (parallel shifts in the vertical direction of the graph) can be included in the time view. However, previous studies have shown that the magnitude of the values affects procrastination^4–8^. Therefore, in this study, to focus on the difference from previous studies, we decided to treat the temporal characteristics, that is, patterns of change along the time axis (graph shape), as the main aspect of the time view. The relationship between the magnitude of stress and the time-series pattern of stress needs to be verified in the future.

In the present study, individual data were categorized using clustering analysis, and it was confirmed that individual data did not always follow the V-shape expressed by the overall mean. This result suggests that to understand and intervene in the complex behavior of procrastination in daily life, it is necessary to analyze not only the overall tendency but also individual differences and provide personalized support. At the same time, since the sample size and area were limited in this study, additional examination is needed in the future to verify the universality of the findings. Also, the participants in our study were limited to only those in their 20 s. Therefore, we need to examine whether the findings of our study apply universally across generations in future studies.

## Method

### Participants

A pooling organization company (Rakuten Research) conducted a web survey. Two filler items (e.g., please respond to this item as “1”) were assigned during the questionnaire to ensure that participants responded attentively and accurately. Data were collected from 296 participants (159 females, M age = 25.6, Range = 20–29 years old, SD = 2.8) who provided informed consent before the experiment and correctly responded to the filler item. Studies examining procrastination by generation have reported that the degree of procrastination varies by age, with younger people being more likely to procrastinate. For this reason, this study focused on people in their 20s^2,26^. The experiment was approved by the research ethics committee of the University of Tokyo and was performed in accordance with the Declaration of Helsinki (approval number: 571–22).

### Measures

#### Procrastination scale

Procrastination level was assessed using 12 items from the Japanese version of the Pure Procrastination Scale^8,27^. At the beginning of the session, the participants were instructed to think about how much each item applied to their main activities, such as schoolwork and work. Example items are “I am continually saying “I'll do it tomorrow.”” and “I often find myself performing tasks that I had intended to do days before.” Participants’ responses were recorded on a 5-point Likert-type scale (ranging from 1 = “very seldom or not true of me” to 5 = “very often true of true of me”). The Cronbach's alpha coefficient was 0.94.

#### Chronological stress view, chronological well-being view

**Figure 5:**
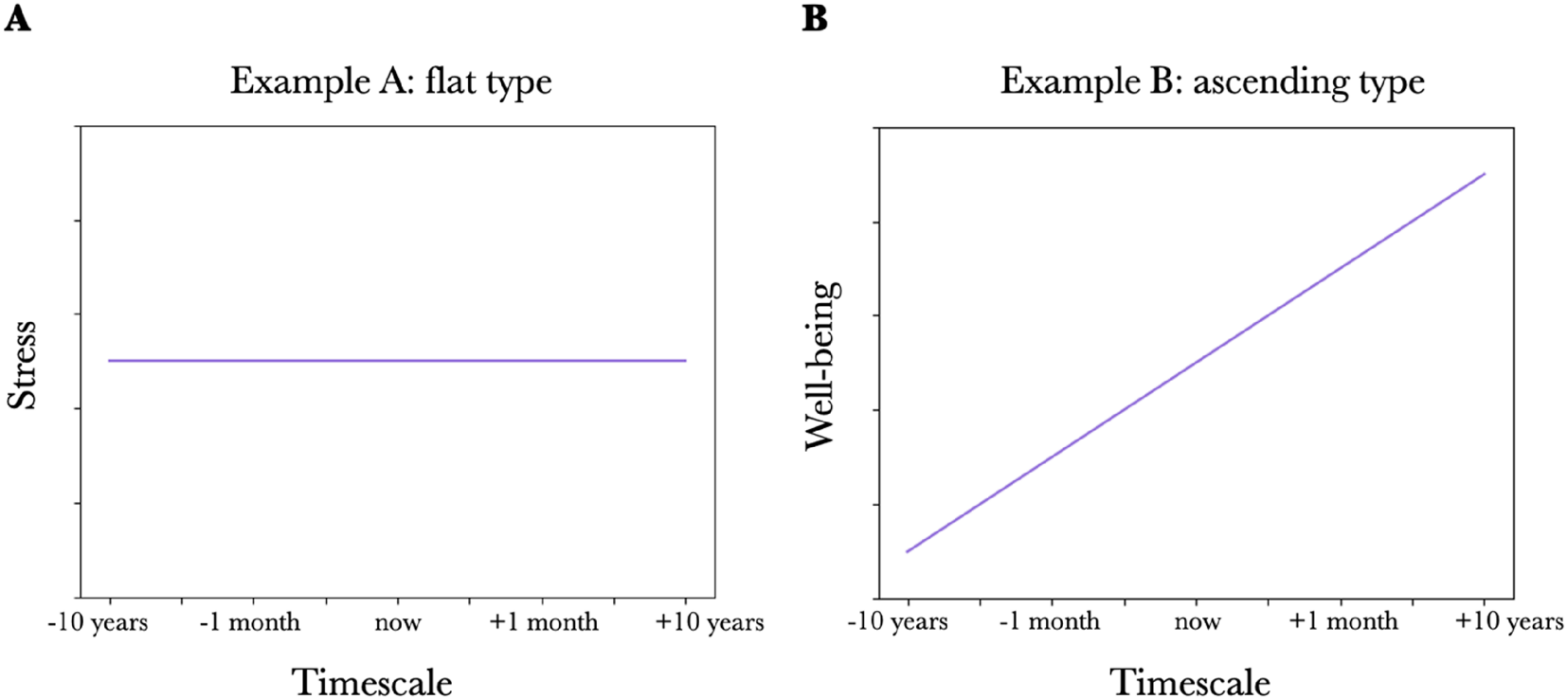
Examples of the chronological stress view, chronological well-being view, The x-axis shows, from left to right, past 10 years, past 1 year, past 1 month, yesterday, now, tomorrow, next 1 month, next 1 year, and next 10 years. The y-axis shows (**A**) stress or (**B**) well-being when asked on the x-axis time axis. (**A**) Example A represents “flat type,” indicating that the value does not change depending on the time axis. (**B**) Example B represents “ascending type,” indicating that the value increases as they move from the past to the present and into the future.

Stress and subjective well-being levels at various time points in the past, present, and future were obtained using the 9-point method (ranging from 1 = "not at all" to 9 = "extremely strong"). Most previous studies asked questions on stress and well-being by specifying a single time axis, for example, "Please think of the past month." In this study, however, the questions were asked along nine-time axes: "the past 10 years," "the past year," "the past month," "yesterday," "now," "tomorrow," "the next month," "the next 1 year," and "the next 10 years.”(Example questions: "How stressed have you felt in the past 10 years?”, “How happy do you feel now?”, “How stressed do you think you will feel in the next year?”) The stress and well-being values obtained from these questions were arranged chronologically on the time axis and defined as the chronological stress view and chronological well-being view, respectively (see Fig. 5 for examples). The definitions of stress and well-being were written at the beginning of the questions to share the images of "stress" and "well-being" among the participants as much as possible. Stress was defined based on the Perceived Stress Scale^28,29^, and well-being was defined based on Subjective Well-being^30,31^. The specific description is as follows.

In the following questions, "stress" and "well-being" are defined as follows.

Stress: Feeling a burden beyond your ability to cope with or feeling that your situation is unpredictable and beyond your control.

Well-being: To feel that.

- you are having warm and trusting relationships with others.
- you find purpose, meaning, and goals in your life.
- you recognize the multiple aspects of self and possess positive attitude toward the self.
- you are self-determined and independent.
- you are able to choose and govern your environment.
- you are continually growing.

### Data analysis

#### Procrastination level grouping

Based on the total Pure Procrastination Scale score, the top 25% of procrastinators (N = 70, Mean = 43.90, SD = 57.80) were defined as “severe procrastinators,” the bottom 25% of procrastination score (N = 88, Mean = 17.18, SD = 8.63) were defined as “low procrastinators,” and the middle 50% of procrastination score (N = 138, Mean = 28.34, SD = 17.13) were defined as “middle procrastinators”.

#### Standardization of the chronological stress and well-being views

When standardizing the individual data for the chronological stress and well-being views, data from subjects with non-zero variance were standardized as usual (mean = 0, variance = 1), while data from subjects with zero variance were standardized only by setting the mean to zero.

#### Hypothesis testing

We hypothesized that the group that holds thoughts that lead to disregard for the future (i.e., stress increases as one moves into the future and well-being decreases as one moves into the future) would be more likely to be severe procrastinators. To test this hypothesis, chronological stress/well-being views were subjected to cluster analysis and classified into several groups based on temporal characteristics, and the proportions of severe procrastinators, middle procrastinators, and low procrastinators in each group were compared.

If our hypothesis is correct, we would expect to find that the groups who believe that stress will decrease in the future and well-being will increase in the future, i.e., those who have an optimistic view of the future, would have a lower percentage of severe procrastinators or a higher percentage of low procrastinators. Also, the group that believes that stress will increase in the future and the group that believes that well-being will decrease in the future, i.e., the group that has a pessimistic view of the future, should have a higher percentage of severe procrastinators or a lower percentage of low procrastinators. To test this hypothesis, the proportions of the severe procrastinators (top 25% procrastination score), middle procrastinators (middle 50%), and low procrastinators (bottom 25%) for each group classified by cluster analysis were calculated and compared using a chi-square test and a residual analysis.

## Conclusion

In this study, we introduced new measures, the chronological stress view and chronological well-being view, and examined their relationship with procrastination. First, we found that the overall mean of chronological stress and chronological well-being views depicted a V-shape, with the present moment as the lowest value. Further analysis showed that the proportion of severe procrastinators was lower in the group that thought stress would not increase in the future (descending type). No relationship was found between chronological well-being and procrastination. These results suggest that people with optimistic thoughts about the future are less likely to be severe procrastinators.

### Supplementary Information

Supplementary Figures.

## Data Availability

The datasets used and/or analyzed during the current study available from the corresponding author on reasonable request.
