# Supplementary material for: Future optimism group based on the chronological stress view is less likely to be severe procrastinators
Source: Sci Rep. 2024 May 30;14:11338. doi: 10.1038/s41598-024-61277-y (PMC11139897; doi:10.1038/s41598-024-61277-y)
Supplement: Supplementary file 1 — Supplementary Figures. [file 41598_2024_61277_MOESM1_ESM.docx]

Supplementary Information


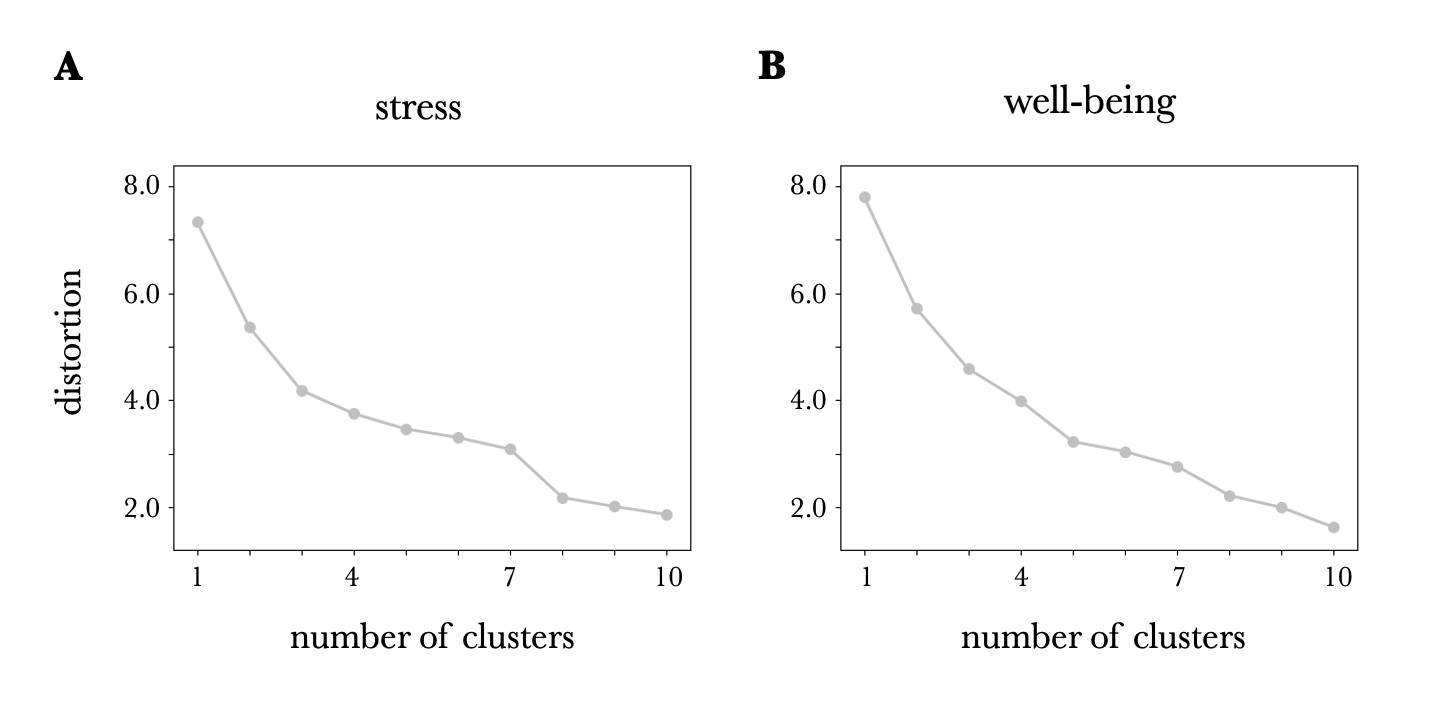


Figure 6. Examination of the number of clusters using the elbow method

(A) for chronological stress view (B) for chronological well-being view. x-axis: number of clusters; y-axis: distortion.
